# Neutrophilia as prognostic biomarker in locally advanced stage III lung cancer

**DOI:** 10.1371/journal.pone.0204490

**Published:** 2018-10-10

**Authors:** Antoine Schernberg, Laura Mezquita, Angela Boros, Angela Botticella, Caroline Caramella, Benjamin Besse, Alexandre Escande, David Planchard, Cécile Le Péchoux, Eric Deutsch

**Affiliations:** 1 Radiation oncology department, Gustave Roussy Cancer Campus, Villejuif, France; 2 INSERM 1030, Gustave Roussy Cancer Campus, Villejuif, France; 3 Department of Medical Oncology, Gustave Roussy, Université Paris-Saclay, Villejuif, France; 4 Department of Radiology, Gustave Roussy, Université Paris-Saclay, Villejuif, France; 5 Université Paris Sud, Université Paris Saclay, Faculté de médecine du Kremlin-Bicetre, Le Kremlin-Bicetre, France; Seoul National University College of Pharmacy, REPUBLIC OF KOREA

## Abstract

**Objective:**

To study the prognostic value of baseline leukocytosis or neutrophiliain two retrospective cohorts of stage III Non-Small Cell Lung Cancer (NSCLC) patients.

**Materials and methods:**

Clinical records of consecutive previously untreated NSCLC patients in our Institution between June 2001 and September 2016 for stage III NSCLC were collected. The prognostic value of pretreatment leucocyte disorders was examined, with focus on patterns of relapse and survival. Leukocytosis and neutrophilia were defined as a leukocyte count or a neutrophil count exceeding 10 and 7 G/L, respectively.

**Results:**

We identified 238 patients, displaying baseline leukocytosis or neutrophilia in 39% and 40% respectively. Most were diagnosed with adenocarcinoma (48%), and stage IIIB NSCLC (58%). 3-year actuarial overall survival (OS) and progression-free survival (PFS) were 35% and 27% respectively. Local relapses were reported in 100 patients (42%), and distant metastases in 132 patients (55%). In multivariate analysis, leukocytosis, neutrophilia, and induction chemotherapy regimen based on carboplatin/paclitaxel were associated with worse OS and PFS (p<0.05). Neutrophilia independently decreased Locoregional Control (LRC) (HR = 2.5, p<0.001) and Distant Metastasis Control (DMC) (HR = 2.1, p<0.001). Neutrophilia was significantly associated with worse brain metastasis control (p = 0.004), mostly in adenocarcinoma patients (p<0.001).

**Conclusion:**

In stage III NSCLC patients, treated with concurrent cisplatin-based chemoradiation, baseline leukocytosis and neutrophilia were associated with worse OS, PFS, LRC, and DMC. In addition with previously available markers, this independent cost-effective biomarker could help to stratify stage III NSCLC population with more accuracy.

## Introduction

Non-small cell lung cancer (NSCLC) represents 83% of patients diagnosed with lung cancer [1]. Despite advances in diagnosis and treatment management, patients prognosis remains poor, with 5-year overall survival (OS) of 27% for early or locally advanced and 4% for metastatic disease [1].

There is a lack of validated prognostic biomarkers in stage-III NSCLC. Tumor stage, performance status, smoking status, age, and gender (PS) are the historical ones [2]. Few are used in clinical practice to guide treatment and determine prognosis. In parallel with -omics recent fields of study, e.g. genomics or proteomics, affordable and easy to access markers have been investigated in NSCLC. Carcinoembryonic antigen (CEA), cytokeratin fragment 21–1 (Cyfra 21–1), and inflammation biomarkers have been found to be associated with patients outcome [3,4]. In metastatic NSCLC, molecular characterization has led to the definition of new subgroups, such as epidermal growth factor receptor (EGFR)-mutated NSCLC, and anaplastic lymphoma kinase (ALK)-rearranged NSCLC, among others that need specific treatments and strategies [5]. Certain mutations are predictive of clinical activity: the tyrosine kinase inhibitors targeting EGFR and ALK have shown striking efficacy in clinical trials, and are currently the standard of care in the clinic, regardless of others risk factors [5]. Still, search for prognostic factors is warranted, especially if they are inexpensive.

Studies revealed relations between systemic inflammation and immunology in the development and progression of various cancers [6]. Neutrophils are the most abundant white blood cells and play a key role in inflammation. They also dominates the immune cell composition in NSCLC [7]. Tumour reactive lymphocyte T cells are also frequently present [7].

The aims of the current study were to assess the clinical utility leukocytosis and neutrophilia in patients diagnosed with stage-III NSCLC, and to compare their accuracy with established prognostic markers.

## Materials and methods

### Patients and tumors

We examined clinical records of consecutive previously untreated and histologically confirmed stage III NSCLC registered in our institution, between June 2001 and September 2016. Only non-operated patients were included, whether with unresectable tumor, surgical contraindications or an impaired performance status. The participants provided consent for their medical records to be used in this research, and data was accessed anonymously. This study was approved approved by Gustave Roussy's Scientific Commission of Clinical Trials (CSET).

All patients had been referred to a multidisciplinary lung tumor board prior to treatment initiation. Explorations at diagnosis included physical examination, endoscopy with biopsy, computed tomography (CT) exploring cervical and thoracic regions, with or without brain magnetic-resonance imaging (MRI) and positron-emission tomography (PET-CT). Disease staging was defined according to the UICC’s lung cancer TNM staging classification, 7th edition.We excluded patients treated in a palliative intent with hypofractionated chemoradiation, patients who received neoadjuvant chemotherapy, patients treated for an immune disease or under steroids, and patients with acute infection defined as defined as the use of antibiotic therapy during the chemoradiotherapy treatment.

In our population, 145 (61%) patients prospectively registered through MSN study (NCT02105168) were included with 136 additional patients. Patients who had chemotherapy before blood count in our institution (n = 16) were excluded from the survival analysis. Patients who had an incomplete set of blood values (n = 27) were excluded as well, leading to 238 patients.

### Complete blood count analysis

Pretreatment blood samples taken in the week preceding the first chemotherapy cycle were used for the current analysis. Leukocytosis and neutrophilia, defining biological inflammation, were defined as blood count ≥ 10 G/L and 7 G/L respectively, while anemia was defined as hemoglobin count < 12.0 g/dL. Thrombocytosis, lymphopenia and monocytosis were defined as platelets count ≥ 400 G/L, lymphocytes count < 1 G/L, and monocytes count ≥ 1 G/L respectively. These cut-off points were chosen because they have been recognized as standard pathological definitions.

### Treatment characteristics and follow-up

All patients had a similar follow-up protocol, with a CT performed every 3 months for the first 2 years, then every 6 months. A PET scan was also performed if there was suspicion of recurrence.

### Statistical analysis

Pearson χ^2^-square test and analysis of variance were used to determine any associations between the variables. Factors associated with tumor relapse were examined. Survival times were defined as the time between the diagnosis and the first event (time of death for OS, time of recurrence or death for PFS, time of loco-regional recurrence LRC, time of distant metastasis for DMC, and time of brain metastasis for brain metastasis control) estimated by the Kaplan Meier method. Patients were censored at the time of the most recent follow-up visit. Survival curves were compared using the log-rank test for the univariate analysis. Multivariate analyses were performed for variables with p value < 0.2 in univariate analysis, according to the Cox proportional hazards model. Neutrophilia and monocytosis were not tested in the same model with leukocytosis, they are subpopulation of leucocytes. Variables with a P value of greater than 0.05 were excluded. Statistical analyses were performed using R (version 3.3.2).

## Results

### Demographics and treatment characteristics

In the population, the median age of the patients at baseline was 60 years (range 25–85). Most were diagnosed with adenocarcinoma (48%), and stage IIIB NSCLC (58%). Most patients received induction chemotherapy (99%), 146 patients (61%) and 71 patients (30%) underwent further chemoradiation or chest radiotherapy alone respectively. Radiotherapy could not be completed in 2 patients due to tumor local progression. No patients underwent surgery following chemoradiotherapy.

At the time of analysis, 157 patients (66%) had died, with a median OS of 19.9 months (range 1.3–148.2). With a median PFS of 10.6 months (range 1.2–138.2), 196 patients (82%) relapsed. Local relapses were reported in 100 patients (42%), and distant metastases in 132 patients (55%). Eighty-one patients (34%) were still alive without evidence of tumor recurrence at time of analysis, with a median follow-up time of 28.4 months (range 1.7–148.2).

The clinicopathological and treatment features are displayed in **Tables 1 and S1.**

**Table 1:** Patients and tumor characteristics.

238 stage-III NSCLC					
Characteristics		Overall population	Neutrophils		
			< 7 G/L	≥ 7 G/L	*p*
		n (%) or median [range]			
**Patients Characteristics**					
**Number**		238	143	95	
**Age**		60 [25, 85]	60 [32, 79]	59 [25, 85]	0.367
**PS**	0	127 (53%)	92 (64%)	35 (37%)	<0.001
	1	101 (42%)	48 (34%)	53 (56%)	
	2	10 (4%)	3 (2%)	7 (7%)	
**Gender**	Male	168 (71%)	96 (67%)	72 (76%)	0.197
	Female	70 (29%)	47 (33%)	23 (24%)	
**Histology**	ADK	114 (48%)	72 (50%)	42 (44%)	0.415
	SCC	48 (20%)	30 (21%)	18 (19%)	
	Other	76 (32%)	41 (29%)	35 (37%)	
**TNM T-status**	T1-2	100 (42%)	70 (49%)	30 (32%)	0.012
	T3-4	138 (58%)	73 (51%)	65 (68%)	
**TNM N-status**	0	19 (8%)	7 (4.9%)	12 (13%)	0.121
	1	8 (3%)	6 (4%)	2 (2%)	
	2	118 (50%)	70 (49%)	48 (51%)	
	3	93 (39%)	60 (42%)	33 (35%)	
**Stage**	III A	101 (42%)	59 (41%)	42 (44%)	0.751
	III B	137 (58%)	84 (59%)	53 (56%)	
**Smoking**	Smoker	213 (90%)	122 (85%)	91 (96%)	0.018
	Active	101 (42%)	85 (59%)	52 (55%)	0.558
**Biology prior Induction CT**					
**Haemoglobin (g/dL**		13.3 [8.3, 17.2]	13.50 [10.50, 17.0]	130 [8.0, 15.90]	0.011
****	< 12 g/dL	44 (19%)	17 (12%)	27 (28%)	0.002
**Platelets (G/L)**		315 [143, 1424]	289 [143, 613]	370 [153, 1424]	<0.001
****	≥ 400 G/L	57 (24%)	19 (13%)	38 (40%)	<0.001
**Leukocytes (G/L)**		9.1 [2.9, 101.7]	7.70 [2.90, 11.70]	12 [8.50, 101.70]	<0.001
****	≥ 10 G/L	93 (39%)	9 (6%)	84 (88%)	<0.001
**Neutrophils (G/L)**		6.2 [1.5, 95.6]	5.0 [1.50, 6.90]	8.70 [7, 95.60]	<0.001
****	≥ 7 G/L	95 (40%)	0 (0%)	95 (100%)	<0.001
**Lymphocytes (G/L)**		1.8 [0.5, 4.4]	1.70 [0.70, 40]	1.80 [0.50, 40]	0.416
****	< 1 G/L	16 (7%)	136 (95%)	86 (91%)	0.264
**Monocytes (G/L)**		0.7 [0.1, 3]	0.60 [0, 1.70]	0.80 [0, 3]	<0.001
****	≥ 1 G/L	47 (20%)	16 (11%)	31 (33%)	<0.001

ADK: adenocarcinoma; CT: chemotherapy; PS: Performance status; SCC: squamous cell carcinoma

### Relationship between baseline leukocytosis or neutrophilia and patient characteristics

On initial blood count, median leukocytes and neutrophils counts were 9.1 G/L (2.9–101.7) and 6.2 G/L (1.5–95.6) respectively. Leukocytosis and neutrophilia were found in 93 (39%) and 95 (40%) patients respectively. Neutrophilia was associated with lower PS (p<0.001). The 8 patients (100%) who did not undergo radiotherapy due to rapid local tumor progression had baseline leukocytosis and neutrophilia.

**Fig 1:**
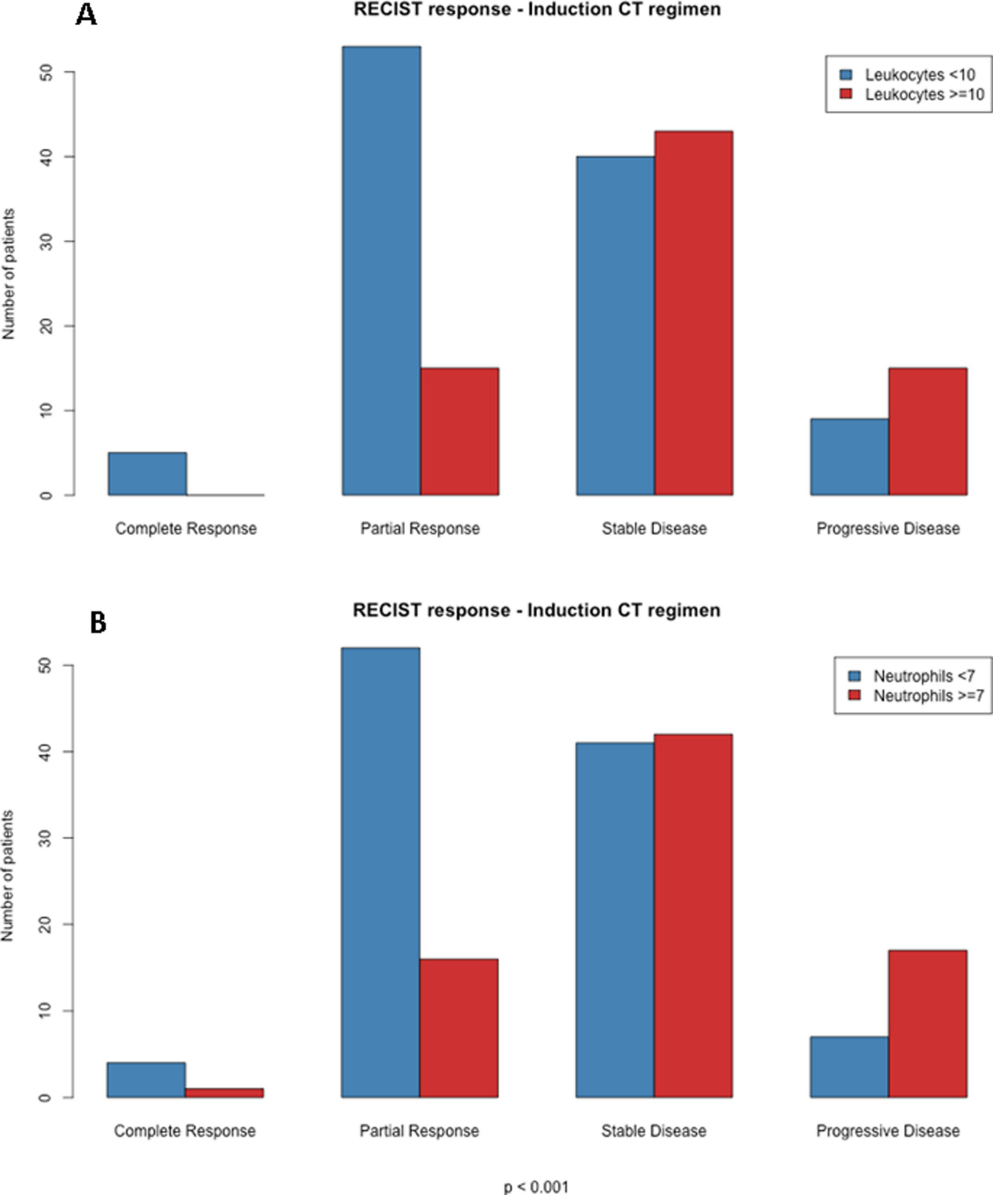
RECIST response assessment after induction chemotherapy in stage-III patients, regarding leukocytosis (A) or neutrophilia (B).

Analyzing total population with 180 assessable responses to induction chemotherapy, there was a correlation between leukocytosis or neutrophilia and increased proportion of patients displaying progression disease or stable disease, vs. partial or complete response (p<0.001, **Fig 1**).

### Impact of leukocyte disorders as predictors of survival

In univariate analysis factors significantly associated with worse OS were leukocytosis (p<0.001), neutrophilia (p<0.001), PS (p = 0.001), TNM-N3 positive status (p = 0.004), stage IIIB vs. IIIA (p = 0.013), non-adenocarcinoma histology (squamous cell carcinoma, others; p = 0.036), concomitant chemotherapy (p = 0.014) and induction chemotherapy regimen others than cisplatin and vinorelbine (p<0.001). The radiotherapy technique (3D vs. IMRT) had no influence on patients’ OS (p = 0.412).

At 3-year follow-up, estimated OS was 44% (95%CI: 36–54%) for patients that had not initial leukocytosis vs. 22% (95%CI: 14–35%) if they had; 3-years PFS was 24% (95%CI: 17–32%) for patients that had not initial leukocytosis vs. 9% (95%CI: 4–19%) if they had. Similarly, estimated 3-years OS was 45% (95%CI: 37–55%) for patients who had not initial neutrophilia vs. 21% (95%CI: 13–34%) if they had and 3-years PFS was 24% (95%CI: 17–33%) for patients that had not initial neutrophilia vs. 8% (95%CI: 4–18%) if they had. Kaplan-Meier curves with univariate analysis regarding leukocytosis or neutrophilia for locoregional control, and distant metastasis control are displayed in **S1 and S2 Figs**. Moreover, analyzing distant relapses, neutrophilia was significantly associated with worse brain metastasis control (p = 0.004), mostly in adenocarcinoma patients (p<0.001) (**S3, S4, S5 and S6 Figs**).

**Fig 2:**
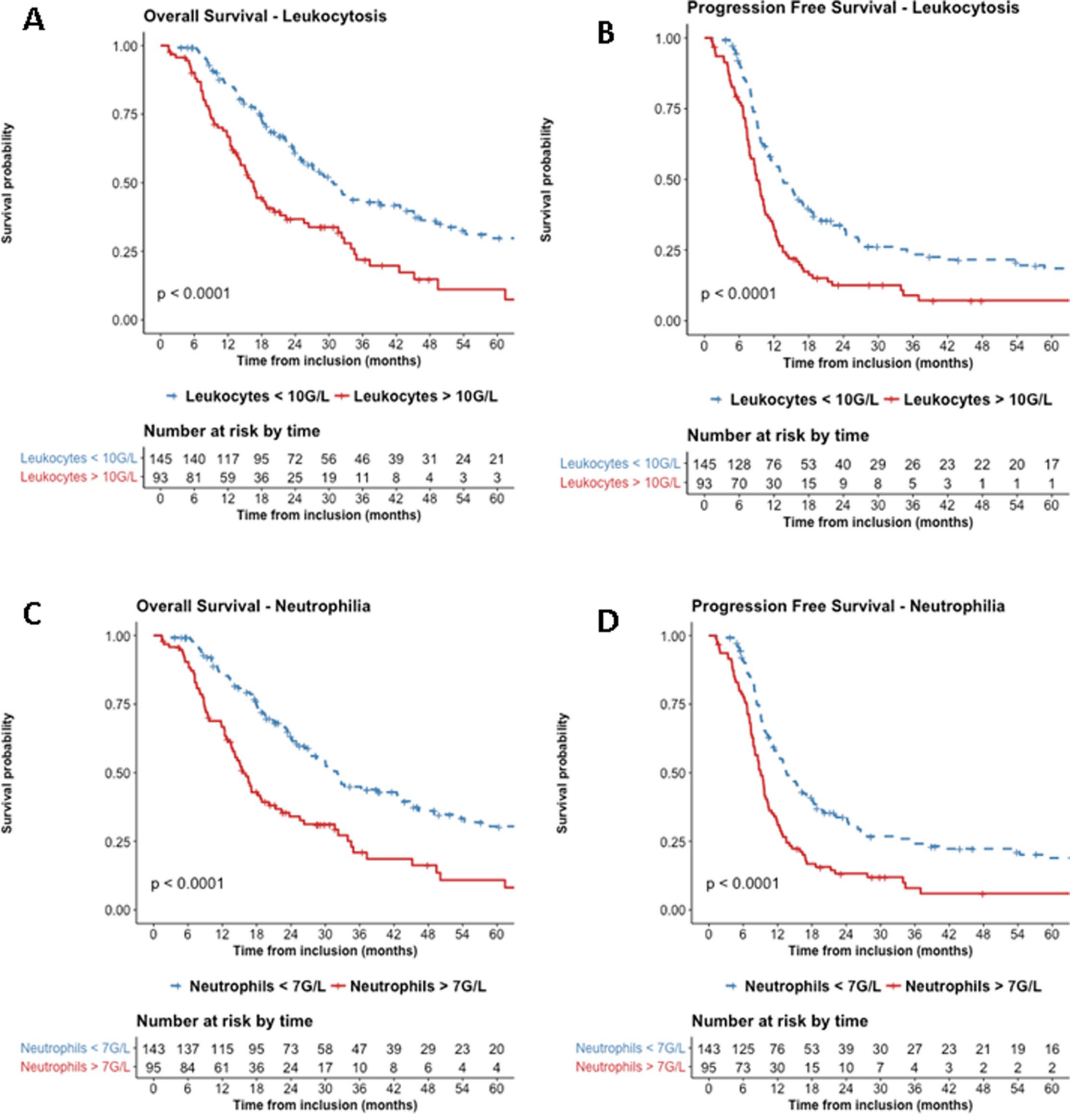
Estimated overall survival in patients with or without leukocytosis (A), progression free survival in patients with or without leukocytosis (B), overall survival in patients with or without neutrophilia (C) or progression free survival in patients with or without neutrophilia (D).

Using multivariate analysis, neutrophilia was independently associated with worse OS with hazard ratio (HR) of 2.3 (95% confidence interval (CI): 1.6–3.3, p<0.001) and worse PFS with HR of 1.9 (95%CI: 1.4–2.6, p<0.001). Similar, leukocytosis was independently associated with worse OS with HR of 2.3 (95%CI: 16–3.3, p<0.001) and worse PFS with HR of 1.8 (95%CI: 1.3–2.5, p<0.001) **(Fig 2 and Table 2)**.

**Table 2:** Results of univariate and multivariate (Cox) analyses for overall survival and progression free survival (significant factors in bold).

Overall population—238 NSCLC stage III patients								
Variable	Overall Survival				Progression Free Survival			
	Univariate log-rank	Multivariate Cox analysis			Univariate log-rank	Multivariate Cox analysis		
	p	HR	95%CI	p	p	HR	95%CI	p
Neutrophilia * (absence)	**< 0.001**	**2.3**	**1.6–3.3**	**< 0.001**	**< 0.001**	**1.90**	**1.4–2.6**	**< 0.001**
Leukocytosis * (absence)	**< 0.001**	**2.3**	**1.6–3.3**	**< 0.001**	**< 0.001**	**1.80**	**1.3–2.5**	**< 0.001**
PS 0 (vs. PS ≥ 1)	**0.001**	**1.5**	**1.0–2.1**	**0.044**	**0.004**	**1.4**	**1.0–1.9**	**0.047**
Age ≥ 65y (vs. <65y)	0.31	—	—	—	0.326	—	—	—
Male gender (vs. Female)	0.545	—	—	—	0.722	—	—	—
Smoker (vs. non-smoker)	0.462	—	—	—	0.61	—	—	—
TNM T3-4 (vs. T1-2)	0.523	—	—	—	0.928	—	—	—
TNM N3 (vs. N0-2)	**0.004**	—	—	0.061	**0.003**	—	—	0.132
Stage IIIb (vs. IIIa)	**0.013**	—	—	0.119	**0.012**	—	—	0.161
Histology ADK (vs. SCC vs. others)	**0.036**	— —	— —	0.130 0.562	0.273	—	—	—
Induction CT regimen CDDP Vinorelbin (vs. Carboplatin Taxol vs. Others)	**< 0.001**	**2.7** **—**	**1.6–4.5** **—**	**< 0.001** **0.272**	**0.002**	**2.0** **—**	**1.3–3.2** **—**	**0.002** **0.177**
Prior surgery (vs. no surgery)	0.266	—	—	—	0.181	—	—	—
Concomitant CT (vs. RT alone)	**0.014**	—	—	0.053	**0.061**	—	—	0.079
RT duration ≥ 50 days (< 50 days)	0.529	—	—	—	0.367	—	—	—
Anemia (absence)	0.953	—	—	—	0.987	—	—	—
Lymphopenia (absence)	**0.096**	—	—	0.737	0.27	—	—	—
Monocytosis * (absence)	0.802				0.586			

*Leukocytosis and subpopulations or derived ratio were not tested in the same model

In multivariate analysis, PS (p = 0.044) and induction chemotherapy other than cisplatin and vinorelbin (p<0.001) were the only features associated with worse survival. Stage IIIB vs. IIIA was not independently related with worse OS or PFS in this population, but was associated with poor DMC. Neutrophilia was also independently associated with worse LRC (HR = 2.5, p<0.001) and DMC (HR = 2.1, p<0.001) (**Table 3)**. Non-adenocarcinoma histologies were independently associated with improved DMC (p<0.05).

**Table 3:** Results of univariate and multivariate (Cox) analyses for locoregional control and Distant Free Metastasis (significant factors in bold).

Overall population—238 NSCLC stage III patients								
Variable	Locoregional Control				Distant Free Metastasis			
	Univariate log-rank	Multivariate Cox analysis			Univariate log-rank	Multivariate Cox analysis		
	p	HR	95%CI	p	p	HR	95%CI	p
Neutrophilia * (absence)	**< 0.001**	**2.5**	**1.6–3.8**	**< 0.001**	**< 0.001**	**2.10**	**1.5–2.9**	**< 0.001**
Leukocytosis * (absence)	**< 0.001**	**2.40**	**1.5–3.7**	**< 0.001**	**0.002**	**2.2**	**1.5–3.2**	**< 0.001**
PS 0 (vs. PS ≥ 1)	**0.001**	**1.6**	**1.0–2.4**	**0.046**	0.222	—	—	—
Age ≥ 65y (vs. <65y)	0.983	—	—	—	0.566	—	—	—
Male gender (vs. Female)	0.388	—	—	—	0.119	—	—	0.10
Smoker active or former (vs. non-smoker)	0.132	—	—	—	0.877	—	—	—
TNM T3-4 (vs. T1-2)	0.444	—	—	—	0.454	—	—	—
TNM N3 (vs. N0-2)	0.067				**0.007**	—	—	0.386
Stage IIIb (vs. IIIa)	0.097	—	—	—	**0.016**	**1.8**	**1.2–2.6**	**0.01**
Histology ADK (vs. SCC vs. others)	0.570	—	—	—	**0.048**	0.66 0.59	0.43–0.99 0.37–0.96	0.048 0.032
Induction CT regimen CDDP Vinorelbin (vs. Carboplatin Paclitaxel vs. Others)	**< 0.001**	3.4 1.7	1.9–6.1 1.1–2.7	< 0.001 0.020	0.481	—	—	—
Prior surgery (vs. no surgery)	0.609	—	—	—	0.656	—	—	—
Concomitant CT (vs. RT alone)	0.293	—	—	—	0.443	—	—	—
RT duration ≥ 50 days (< 50 days)	0.507	—	—	—	0.353	—	—	—
Anemia (absence)	0.674	—	—	—	0.853	—	—	—
Lymphopenia (absence)	0.141	—	—	0.674	0.862	—	—	—
Monocytosis * (absence)	**0.078**				0.761			

*Leukocytosis and subpopulations or derived ratio were not tested in the same model.

Anemia: < 13 g/dL; CDDP: cisplatin; Leukocytosis: leukocyte count ≥ 10 G/L before induction CT; Neutrophilia: neutrophil count ≥ 7 G/L before induction CT;

## Discussion

Several histopathological features and molecular biomarkers have been studied as potential prognostic factors. Still, in locally-advanced NSCLC patients, the only ones which are commonly used in clinical decision-making are patient’s performance status and tumour stage. In our study we hypothesized that a simple biological feature such as neutrophilia could be prognostic in locally advanced NSCLC. Both leukocytosis and neutrophilia were associated with poor OS and PFS, in a large set of patients. Moreover, there was a strong association between neutrophilia, LRC and poor response to induction chemotherapy, when evaluated.

Tumor-related leukocytosis and neutrophilia, established after excluding obvious causes (infection, bone marrow metastasis, and corticosteroid use), may result from hematopoietic colony-stimulating factors and inflammatory cytokines direct from solid tumors, comprising granulocyte colony-stimulating factor (G-CSF) among others [8,9]. Since the first report of G-CSF-producing malignant lung cancer in 1977, studies in cervical, head and neck, lung, gastric, bladder…, among others, described this particular biological characteristic, related with patient outcome [10–12].

Myeloid-derived suppressor cells (MDSCs) expands during cancer and inflammation, associated with a remarkable ability to suppress T-cell responses [13]. Debate persists as to which these suppressive properties are maintained by their tumour-infiltrating counterparts [14]. MDSCs both can exert antitumoral or protumoral activity. TGF-β within the tumor microenvironment induces a population of Tumor-Associated Neutrophils (TANs) with a protumor N2 phenotype [15]. TGF-β blockade slows tumor growth through activation of CD8+ T cells, macrophages, and TANs with an antitumor N1 phenotype [15]. Radiation therapy may also induces the N1 polarization [16]. A flow cytometry panel measuring 51 immune cell populations recently determined neutrophils as the most prevalent immune cell type in NSCLC microenvironment, that could partially explains why ~80% of NSCLC patients initially fail immune checkpoint inhibitor therapy [7]. Moreover, the neutrophil (and not the monocyte) population negatively correlated with CD8+ content, while increased levels CD8 + tumor-infiltrating lymphocytes (TILs) have been previously associated with better outcome in NSCLC [7,17]. In the present study, local relapses were reported in 100 patients (42%), and distant metastases in 132 patients (55%); both leukocytosis and neutrophilia decreased locoregional control (p<0.001).

There are evidences that neutrophils protect tumor cells through metastasis process [18]. Neutrophils facilitate intermediate steps of the invasion-metastasis cascade, suppressing natural killer cell activity and enhancing the extravasation of tumor cells, mainly through the secretion of various matrix metalloproteinases (MMPs) [19]. Such protection from attack by innate and adaptive immune system offers a clear advantage to tumor cells in transit [20]. Neutrophil NETosis, the formation of Neutrophil extracellular traps (NETs), is a unique form of cell survival mechanism characterized by the release of networks of extracellular fibers primarily composed of DNA from neutrophils which entangle pathogens [20,21]. Such entangled circulating tumor cells may be more apt to survive intraluminally, adhere to endothelial cells, and extravasate [20]. NETs were displayed as potential candidate pharmaceutical targets in cancer patients [21]. In accordance with these findings, in the present study neutrophilia independently decreased DMC (p<0.001). Moreover, prior study recently associated elevated neutrophil to lymphocyte ratio (NLR) with poor brain metastasis control, mostly in adenocarcinoma patients [22]. In accordance, the present study displayed similar findings, with neutrophilia decreasing brain metastasis control, mostly in adenocarcinoma patients.

To translate a given neutrophilia into a personalized prognosis or treatment plan is challenging [23]. Prospective longitudinal measurements of white blood count in individual patients is mandatory to maximize the clinical utility of systemic neutrophil scores [23]. Thus far, a limited number of studies have attempted this approach. Characterization of neutrophil polarization in different tumour types and stages is needed to maximize their prognosis significance and utility as potential therapeutic modalities [23]. Another question is whether parallel scoring of patient serum levels of neutrophil-activating and polarizing soluble mediators (IL-1β, IL-17, G-CSF, GM-CSF and/or TGFβ) increases the prognostic or predictive power of neutrophil measurement [23].

Neutrophil-targeting agents are being developed for the treatment of inflammatory and autoimmune diseases [23]. In patients with chronic obstructive pulmonary disease, CXCR2 antagonist decreases absolute neutrophil counts, reduces biological inflammation and disease symptoms [24]. Inhibition of CXCL8–CXCR1/2 signaling by CXCL8 antibodies, or small molecules targeting CXCR1 and/or CXCR2, also decreases tumor growth and progression in tumor mouse models [25]. CXCR2 inhibition in a preclinical metastatic breast cancer model enhanced response of both tumor and micrometastases to chemotherapy treatment [26]. Clinical trials evaluating reparixin, a CXCR1 and CXCR2 inhibitor, are ongoing in cancer patients [27]. Inhibitors of NE are also being tested and have shown some promise in mouse models of lung cancer [28] Another neutrophil-associated pathway under intense investigation is the IL-23 –IL-17 axis [23]. Inhibition of the leukotriene-generating enzyme arachidonate 5-lipoxygenase (Alox5) abrogates neutrophil pro-metastatic activity and consequently reduces metastasis [29]. Targeting neutrophil Alox5 inhibition have been reported to limit metastatic progression in preclinical models [29].

A promising therapeutic approach is the combination of T cell checkpoint inhibitor immunotherapy with neutrophil manipulation [23]. Experimental studies have shown that anti-programmed cell death protein 1 (PD1) and anti-cytotoxic T lymphocyte-associated antigen 4 (CTLA4) synergizes with anti-CXCR2 or anti-Ly6G, respectively, to delay tumour growth [30,31]. However, targeting neutrophils can be associated with side effects as neutrophils are critical for host defense against infection [25]. Alternative approach is the idea that neutrophil resolution of inflammation could be induced within the tumor microenvironment, possibly resolved by neutrophil reverse migration rather than whole neutrophil targeting all circulating neutrophils [25].

Finally, not all neutrophils have protumor effects. Tumor associated N2 neutrophils are characterized by high expression of CXCR4, VEGF, and gelatinase B/MMP9 and can be induced on exposure to high TGF-β levels [25]. By contrast, N1 neutrophils are induced on TGF-β blockade and express immunoactivating cytokines and chemokines and low levels of arginase and are able to kill cancer cells [25]. Understanding how neutrophils are polarized and if and how they can be reprogrammed will be crucial to developing successful cancer therapies [25]. TGF-β inhibitors in oncology have therefore moved towards the use of combinatorial therapies with considerable promise for the clinic [32]. Clinical trials are ongoing [33].

The present study is one of the largest locally-advanced cohort associating both leukocytosis and neutrophilia with OS in NSCLC to our knowledge [34,35]. The relatively small sample size and the retrospective nature of our study, despite a prospective patients registration through MSN study (NCT02105168), should be acknowledged as potential limitations. In addition, although there is a postulated pathological inflammatory mechanism in carcinogenesis, infection and concomitant corticosteroids intakes alters the prognostic utility of these parameters. However, the process of validation in a second independent case series, in addition with independent metastatic cohort, supports baseline leukocyte and neutrophil absolute counts as predictors of survival in patients with stage-III NSCLC.

## Conclusion

This study recognized baseline leukocytosis and neutrophilia to be independent prognosis markers for OS, PFS and DMC in stage-III NSCLC. This accessible biomarker may guide patients' management.

## Supporting information

S1 Table Treatment characteristics. (DOCX)

S1 Fig Estimated locoregional control in patients with or without leukocytosis. (TIF)

S2 Fig Estimated locoregional control in patients with or without neutrophilia. (TIF)

S3 Fig Estimated distant metastasis control in patients with or without leukocytosis. (TIF)

S4 Fig Estimated distant metastasis control in patients with or without neutrophilia. (TIF)

S5 Fig Estimated brain metastasis control in patients with or without neutrophilia. (TIF)

S6 Fig Estimated brain metastasis control in patients with or without neutrophilia, in adenocarcinoma patients. (TIF)

S1 File Data lung.csv. Anonymized analyzed data.
(CSV)
