# Supplementary material for: Neutrophilia as prognostic biomarker in locally advanced stage III lung cancer
Source: PLoS One. 2018 Oct 10;13(10):e0204490. doi: 10.1371/journal.pone.0204490 (PMC6179235; doi:10.1371/journal.pone.0204490)
Supplement: S1 Table — (DOCX) [file pone.0204490.s001.docx]

**S1 TABLE. Treatment characteristics.**

| **238 stage-III NSCLC** | | | | | |
| --- | --- | --- | --- | --- | --- |
| **Characteristics** | | **Overall population** | **Neutrophils** | | |
|  |  |  | **< 7 G/L** | **≥ 7 G/L** | *p* |
|  |  | **n (%) or median [range]** | | | |
| **Induction chemotherapy** | | | | | |
| **Number** | | 238 | 143 | 95 |  |
| **Surgery prior** | | 25 (11%) | 19 (13%) | 6 (6%) | 0.133 |
| **Induction CT** | | 237 (99%) | 143 (100%) | 94 (99%) | 0.837 |
| **Regimen** | Cisplatin + Vinorelbine | 125 (53%) | 74 (51.7%) | 51 (54%) | 0.255 |
|  | Carboplatin + Taxol | 34 (14%) | 19 (13%) | 15 (16%) |  |
|  | Other | 79 (33%) | 50 (35.0%) | 29 (30%) |  |
| **N cycles** | | 2 [1, 7] | 2 [1, 7] | 2 [1, 6] | 0.179 |
|  | 1-2 cycle | 127 (55%) | 75 (52%) | 52 (55%) | 0.57 |
|  | >2 cycles | 108 (45%) | 67 (46.9%) | 41 (43%) |  |
| **RECIST evaluation after induction** | |  |  |  |  |
|  | Progressive Disease | 11 (5%) | 7 (4.9%) | 17 (18%) | <0.001 |
|  | Stable Disease | 83 (35%) | 41 (28.7%) | 42 (44%) |  |
|  | Partial Response | 68 (29%) | 52 (36%) | 16 (17%) |  |
|  | Complete Response | 5 (2%) | 4 (3%) | 1 (1%) |  |
|  | Not assessable | 58 (24%) | 39 (27%) | 19 (20%) |  |
| **Radiotherapy** | | | | | |
| **Concomitant CT** | | 146 (61%) | 87 (60.8%) | 59 (62%) | 0.952 |
| **RT alone** | | 71 (30%) | 48 (34%) | 23 (24%) | 0.161 |
| **Regimen** | Cisplatin + Vinorelbine | 106 (45%) | 60 (42%) | 46 (48%) | 0.376 |
|  | Carboplatin + Paclitaxel | 15 (6%) | 10 (7%) | 5 (5%) |  |
|  | Other | 22 (9%) | 16 (11%) | 6 (6%) |  |
| **N concomitant CT cycles** | | 2 [0, 7] | 2 [0, 7] | 2 [0, 7] | 0.201 |
| **RT duration (days)** | | 48 [11, 91] | 48 [11, 71] | 48 [13, 91] | 0.877 |
| **RT duration ≥ 50 days** | | 56 (24%) | 33 (23%) | 23 (24%) | 0.004 |
| **RT dose (Gy)** | | 66 [30, 70] | 66 [30, 70] | 66 [30, 70] | 0.38 |
| **RT number of fractions** | | 33 [10, 55] | 33 [10, 55] | 33 [10, 35] | 0.27 |

*CT: chemotherapy; N: number ratio; NA: not applicable; PLR: Platelet to lymphocyte ratio; RT: radiotherapy;*
